# Multimodal MRI combined with RNA sequencing reveals pathological signatures in the 9-month-old 3×Tg-AD mouse brain

**DOI:** 10.4103/NRR.NRR-D-25-00006

**Published:** 2025-09-29

**Authors:** Yongxin Li, Ziling Tang, Maohua Yao, Yun Ran, Zuocheng Qiu

**Affiliations:** 1 Guangzhou Key Laboratory of Formula-pattern Research Center, School of Traditional Chinese Medicine, Jinan University, Guangzhou, Guangdong Province, China; 2 Guangzhou Provincial Key Laboratory of Speed Capability Research, Jinan University, Guangzhou, Guangdong Province, China

**Keywords:** 3×Tg-AD mouse model, Alzheimer’s disease, brain volume, brain–behavior correlation, diffusion tensor imaging, fractional anisotropy, magnetic resonance imaging, nerve regeneration, RNA sequencing, voxel-based morphometry

## Abstract

The triple transgenic mouse model of Alzheimer’s disease (3×Tg-AD) is a widely used model that exhibits region-dependent patterns of progressive amyloid-β and tau pathology. Although structural brain abnormalities on magnetic resonance imaging have been observed in 3×Tg-AD mice at later disease stages (> 12 months) and as early as 2 months, few studies have investigated changes in these mice during the stage with extensive amyloid-β deposition and onset of tau pathology (around 9 months). This study aimed to assess brain morphometry and microstructure alterations in 9 month-old 3×Tg-AD mice to better understand the neural mechanisms underlying these specific pathological features. Voxel-based analyses were employed on T2-weighted and diffusion tensor imaging to identify differences between 3×Tg-AD and control mice. Compared with controls, 3×Tg-AD mice exhibited lower gray matter volume in several regions including both hippocampal regions, the right thalamus, the left caudoputamen, and the cortex. Reduced white matter volume was observed in fiber tracts including the corpus callosum, internal capsule, stria terminalis, and olfactory tract. Whole-brain diffusion tensor imaging analysis revealed a significant decrease in fractional anisotropy and an increase in both radial and mean diffusivity within the left dentate gyrus of the hippocampal region and right striatum-like amygdala nuclei, with no significant difference in axial diffusivity. Correlation analyses demonstrated significant associations between behavioral performance measures, with both gray and white matter volumes within regions showing significant morphometric differences. Notably, behavioral performance also exhibited significant correlations with diffusion tensor imaging measures particularly within the left dentate gyrus of the hippocampal region and right striatum-like amygdala nuclei. Immunofluorescence analysis confirmed increased amyloid-β plaques and p-Tau protein expression in the hippocampal regions of 3×Tg-AD mice, which corroborated the magnetic resonance imaging findings. Transcriptome analysis in hippocampus tissue identified 1389 differentially expressed genes. Gene Ontology and Kyoto Encyclopedia of Genes and Genomes pathway analyses revealed that numerous differentially expressed genes were enriched in biological processes relevant to synapse structure, cognition, learning, and memory, with particular emphasis on Wnt and mitogen-activated protein kinase signaling pathways. Collectively, these findings suggest that intricate anatomical and microstructural alterations occur in 3×Tg-AD model mice at the onset of pathology around 9 months, potentially driven by gene expression alterations. Moreover, our results support the potential utility of brain volume and diffusion metrics as biomarkers for Alzheimer’s disease pathology, which could have significant implications for clinical diagnosis of Alzheimer’s disease patients.

## Graphical abstract

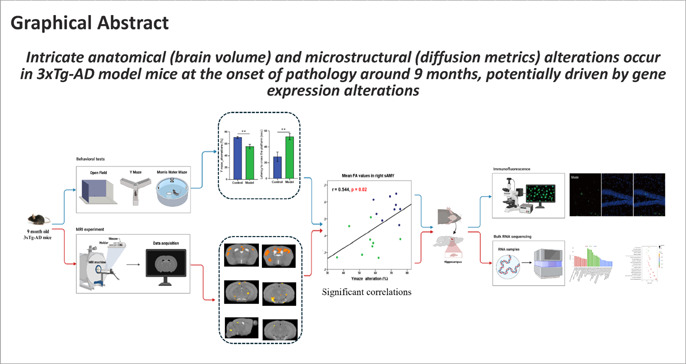

## Introduction

Alzheimer’s disease (AD) is the most prevalent form of dementia. Patients with this disorder present a spectrum of clinical symptoms, including age-related memory decline, cognitive impairment, and difficulties in executing complex daily activities (Kamiya et al., 2018). Previous studies of AD pathology have demonstrated that the disease is characterized by the presence of neuritic plaques and neurofibrillary tangles resulting from amyloid-beta (Aβ) accumulation, aggregation of fibrillar plaques, and hyperphosphorylation of tau protein (Depp et al., 2023; Zhang et al., 2023; Kovacs et al., 2024; Wang et al., 2024; Zhang et al., 2025). Consistent with these pathological processes, decreased synaptic density, impaired glucose metabolism, neuronal loss, and disruption in white matter (WM) are essential pathological features observed in patients with AD (Sharp et al., 2023). Despite extensive research efforts into understanding AD pathology, the causative factors contributing to its progression remain unclear.

Currently, the primary focus in AD research is to identify diagnostic biomarkers for disease development and progression. Given that AD is a slowly progressive neurodegenerative condition, different stages of disease development correspond to distinct pathological and structural changes in affected individuals. Recent advances in diagnostic imaging techniques have provided valuable insights into the temporal course of AD pathology both in clinical patients and animal models (Dubois et al., 2021; Ni, 2021; Zeng et al., 2021a). Abnormal protein accumulation is believed to play a central role in the neurodegenerative alterations observed. Previous studies have demonstrated that noninvasive imaging techniques can provide opportunities to monitor the progression of neurodegenerative diseases and assess treatment response (Chen et al., 2023, 2024; Dong et al., 2024). Positron emission tomography and magnetic resonance imaging (MRI) are commonly used in both preclinical research and clinical diagnosis (Dubois et al., 2021; Insel et al., 2022; Jullienne et al., 2022; Aberathne et al., 2023; He et al., 2025). Diffusion MRI is the foremost innovative imaging technique, as it can provide distinct information regarding neuronal tissue microstructure and composition (Devanarayan et al., 2024). Numerous clinical studies have confirmed the presence of cortical microstructural alterations across the clinical continuum from mild cognitive impairment to advanced stages of AD (Vogt et al., 2020; Stone et al., 2021). Structural MRI has directly observed brain atrophy, which holds potential for early AD diagnosis (Brueggen et al., 2015; Huang et al., 2023). Owing to its ability to identify characteristics such as cerebral volume changes and microstructural injury, brain MRI provides valuable information reflecting the underlying neural mechanisms of AD. Although previous studies have yielded important results (Vogt et al., 2020; Stone et al., 2021; Huang et al., 2023), these findings may have been influenced by factors such as genetic differences, subject variables, and certain investigative techniques that cannot be applied to humans (e.g., ultra-high field MRI) (McGraw et al., 2017). Animal models have shown great promise in studying AD, particularly through isolating specific gene manipulations (Martini et al., 2018; Humpel and Foidl, 2020).

One commonly used animal model of AD is the triple transgenic mouse model (3×Tg-AD), which possesses three gene mutations associated with human familial AD: the human presenilin-1 M146V mutation, human amyloid precursor protein Swedish mutation, and human tau protein P301L mutation (Chen et al., 2023; Li et al., 2025). This model exhibits both Aβ accumulation and neurofibrillary tangles following a temporal and spatial pattern similar to that observed in human AD (Bloom, 2014; Belfiore et al., 2019). In addition, the age-related pathological cascade has been studied in this model, which has the following characteristics: intraneuronal Aβ begins accumulating around 2 months of age while extracellular Aβ deposits become apparent at 6 months. By 12 months of age, Aβ deposits have spread extensively from the caudal hippocampus to other regions (Belfiore et al., 2019). Tau pathology becomes detectable around 6 months of age, and neurofibrillary tangles appear after 12 months (Belfiore et al., 2019). Cognitive impairment manifests at the age of 3 to 4 months. Alongside the progression of AD pathology, age-related brain atrophy and myelin loss are observed on advanced high-field MRI (Ni, 2021; Jullienne et al., 2022; Bergamino et al., 2023). Several studies using diffusion tensor imaging (DTI) on the 3×Tg-AD mouse model have identified significant changes in DTI metrics (both increases and decreases) within the hippocampus in mice between 2 and 14 months of age (Snow et al., 2017; Manno et al., 2019; Falangola et al., 2020). Similar DTI changes have also been reported in other regions such as the fimbria, basal forebrain, external capsule, and internal capsule, characterized by decreased fractional anisotropy (FA) and axial diffusion (AxD) and increased radial diffusion (RD) (Falangola et al., 2020; Falangola et al., 2021; Ni, 2021; Bergamino et al., 2023). However, a previous DTI study did not observe any changes in diffusion metrics or alterations in myelin staining or WM volumes across mice aged from 11 to 17 months (Kastyak-Ibrahim et al., 2013). The inconsistent findings among these DTI studies suggest that age-related diffusion metrics changes in the 3×Tg-AD mouse model are complex (Nie et al., 2019). In addition to tissue microstructure alterations, brain-wide volume measurements have also yielded inconsistent results. Although most studies have reported volumetric changes throughout various brain regions over time (including both decreases and increases) (Kong et al., 2018; Chiquita et al., 2019; Rollins et al., 2019; Güell-Bosch et al., 2020), others have found no significant differences in brain volume between 3×Tg-AD mice and controls (Kastyak-Ibrahim et al., 2013; Wu et al., 2015; Montoliu-Gaya et al., 2018). These inconsistent results may be attributed to various factors. Several longitudinal studies have aimed to elucidate this temporal change (Chen et al., 2023; Khodadadi Arpanahi et al., 2024). In comparison with control observations, the trajectory of volumetric change in the bilateral hippocampi, entorhinal cortex, and frontal regions, and fimbria exhibited an “inverted-U” shape in AD mice (Kong et al., 2018). The observed neuroanatomical and microstructural changes in these studies are likely consequences of pathophysiological processes. These prior neuroimaging investigations indicate that MRI can detect neuroanatomical and microstructural alterations in the 3×Tg-AD mouse model throughout its lifespan. However, most previous neuroimaging studies were conducted at later stages (Kastyak-Ibrahim et al., 2013; Snow et al., 2017; Falangola et al., 2022) or as early as 2 months of age (Manno et al., 2019; Falangola et al., 2020). Few MRI studies have explored imaging changes in this model during the stage when extensive Aβ deposition begins and tau pathology emerges (around 9 months). It is at this specific stage that neuroinflammation becomes evident and Aβ deposition shifts from intraneuronal to extracellular (Belfiore et al., 2019). Selecting 9-month-old 3×Tg-AD mice for research not only circumvents the issue of using young mice without obvious pathological characteristics, but also avoids the experimental interference brought about by other age-related diseases in elderly mice (> 12 months old). Additionally, functional MRI can capture the dynamic process of brain reorganization or collapse, offering significant clues for comprehending the disease mechanism. From the perspective of early intervention strategies, mice of this age group typically commence to exhibit cognitive function and behavioral disorders. Research on mice at this stage can test whether early intervention measures can reverse the abnormal functions during the golden window of early intervention. Therefore, examining brain imaging alterations during this stage can provide valuable insights into the pathological mechanisms of AD and identify biomarkers that reflect this particular phase of pathology transformation.

In this study, we aimed to identify neuroanatomical and microstructural changes in 9-month-old 3×Tg-AD mice and determine whether these alterations are indicative of cognitive impairment. To achieve this, we employed voxel-based morphometry (VBM) technology on T2-weighted imaging and calculated diffusion metrics using DTI to evaluate anatomical and microstructural differences between 9-month-old 3×Tg-AD mice and wild-type control mice. VBM is an automated technique that allows for the examination of brain anatomy differences between groups at a voxel-wise level without requiring predefined regions of interest (ROIs) (Good et al., 2001). DTI is a powerful noninvasive method widely used for detecting brain microstructural abnormalities associated with AD (Kantarci et al., 2017; Falangola et al., 2020; Bergamino et al., 2023). The combination of these methods may provide complementary insights into brain changes in AD mouse models. Furthermore, correlation analyses were conducted between the neuroimaging results and behavioral performance to investigate whether neuroimaging changes can reflect levels of behavioral impairment.

Furthermore, the complete elucidation of the regulatory mechanism underlying brain imaging alterations at the molecular level remains elusive. RNA sequencing (RNA-seq) is an effective method for serial analysis of gene expression, which aids in understanding regulation mechanisms (Lachmann et al., 2018). A previous study has used RNA-seq to comprehend the molecular mechanisms underlying AD (Zeng et al., 2021b). The mechanisms responsible for brain structural changes in AD are also poorly understood. Investigating the molecular mechanism behind differences in brain structural alterations between AD and control mice would provide a theoretical basis for comprehending the neural mechanism of AD. To achieve this objective, we further employed RNA-seq to comprehensively analyze and compare gene expression profiles within regions exhibiting consistent neuroimaging differences. Prior research has demonstrated that gene expression distribution can be mapped onto functional MRI (Lachmann et al., 2018). In a previous study that combined genome-wide transcriptional profiling of the insular cortex of 3×Tg-AD mice and control littermates with biochemical and behavioral profiling, wider transcriptional changes triggered by the abnormal deposition of Aβ and neurofibrillary tangles occurred well before behavioral decline, and genes specifically associated with abnormal Aβ protein deposition and cognitive decline were identified (Yin et al., 2020). Such previous studies have demonstrated the feasibility of combining imaging and genomics analysis, which could lead to a better understanding of the behavioral and pathological changes in AD. In this study, we integrated measures of brain structure with genetic transcriptional data to establish how neurobiological processes converge to contribute to AD at 9 months. This design offers novel insights into the neurobiology of AD.

## Methods

### Alzheimer disease mice

The experimental procedures were approved by the National Institutional Animal Care and Ethical Committee (approval No. IACUC-20231102-21) of Jinan University on November 1, 2023. All protocols adhered to the National Institutes of Health Guide for the Care and Use of Laboratory Animals (8^th^ ed., National Research Council, 2011).

To avoid the confounding effects of fluctuating ovarian hormones in females (Beery and Zucker, 2011), nine male 3×Tg-AD mice on a C57BL/6J-congenic background (approximately 7 months old) and nine control mice weighing approximately 25 to 30 g were procured from Wuhan Youdu Biotechnology Company (Wuhan, China; license no. SCXK (E) 2021-0025). The control mice were wild type non-transgenic SPF-grade animals of the same sex, age, and weight as the 3×Tg-AD mice. All animals were housed in a certified animal care facility with controlled temperature (25°C) and 75% humidity, and were exposed to a 12-hour light/dark cycle (lights on at 8:00 a.m.).

### Behavioral tests

The behavioral tests were conducted by two trained and experienced observers who were blinded to the treatment conditions. All animals in the model and control groups underwent behavioral testing at approximately 9 months of age.

#### Open field test

The open field test was used to evaluate locomotor ability (Võikar and Stanford, 2023). The mice were placed in the center of a 50-cm × 50-cm × 40-cm open field box and permitted to move freely for 10 minutes while being observed by a computer-controlled video tracking system (EthoVision 14.0 software; Noldus Information Technology, Leesburg, VA, USA). The total distance each mouse moved was recorded and analyzed.

#### Y-maze test

The Y-maze test was used to assess working memory by measuring spontaneous alternation between arms of the maze (Kraeuter et al., 2019). The Y-maze consisted of three arms positioned at 120° angles and were constructed from light gray plastic material. Each arm was 6 cm in width and 36 cm in length; the walls measured 12.5 cm in height. After each mouse’s exploration period, which lasted 8 minutes, the maze was cleaned using a 70% ethanol solution. The behavior of each mouse was monitored, recorded, and analyzed using Viewer software (EthoVision 14.0 software). A mouse was considered to have entered an arm when its entire body except for its tail had entered an arm; exit occurred when only its tail remained within. An alternating trial was counted if an animal consecutively entered three different arms. Because the maximum number of triads (three successive alternating arm entries) is equal to the total number of arm entries minus two, the spontaneous alternation score was calculated as follows: (number of alternating triads)/(total number of arm entries – 2) × 100. Each test was recorded using a video camera to determine the sequence of entries, enabling identification of spontaneous alternations through three consecutive sets of different arm choices. The alternations of two groups were analyzed.

#### Morris water maze test

The Morris water maze (MWM) test was used to assess spatial learning and memory ability (Vorhees and Williams, 2006). A black cylindrical tank (diameter, 1.20 m; height, 0.4 m) was filled with water which was made to be opaque by adding titanium dioxide. The tank was divided into four equal quadrants and an invisible 10 cm platform was placed in the center of the first quadrant. Mice were trained to find the invisible platform whose surface was 1.5 cm beneath the water for 6 days. During the place navigation test, each mouse was allowed to find the platform for 60 seconds. When the platform was found, the latency of escape was recorded. If the mouse could not find the platform within 60 seconds, it would be manually placed on the platform and remain for 30 seconds. On the seventh day, the platform was removed for the spatial probe trial. The mice were allowed to swim freely for 60 seconds. The latency to cross the platform, number of times it crossed the platform, and the time spent in the target quadrant were recorded and analyzed by a computer-controlled video tracking system (EthoVision 14.0 software). The escape latency of the training test and probe test, duration spent within the target quadrant, and frequency of crossing the target platform were automatically recorded and analyzed.

### Magnetic resonance imaging acquisition

After behavioral testing, all mice were anaesthetized using inhalational isoflurane (Ruiwode Life Science and Technology Co., Shenzhen, China) at a ratio of 1% oxygen and 3% isoflurane during pilot scanning and 0.5% oxygen and 2.5% isoflurane during data acquisition. Then, mice were fixed on the animal bed to minimize head movement by an MRI-compatible cradle with surface and body coils. An animal monitoring unit (Small Animal Instruments Inc., New York, NY, USA) was used to record heart rate and respiratory frequency. Normal body temperature was maintained using a hot water circulation system.

The *in vivo* MRI experiments were all performed on a 9.4 T small animal MRI scanner (Biospec 94/30; Bruker Biospin, Ettlingen, Germany) running ParaVision version 360.1.1 (Bruker Biospin). T2-weighted images were acquired using a Turbo rapid acquisition with relaxation enhancement (TurboRARE) sequence: repetition time = 2500 ms, echo time = 33 ms, matrix size = 256 × 256 × 17, average = 2, spatial resolution = 0.08 × 0.08 × 1 mm^3^, slice thickness = 0.7 mm, and flip angle = 180°. The DTI data were acquired using a SE-EPI diffusion MRI sequence. The main imaging parameters were repetition time = 3000 ms, echo time = 20.7 ms, diffusion gradient duration = 2.5 ms, gradient separation = 8.5 ms, slice thickness = 0.7 mm, matrix size = 108 × 96 × 17, spatial resolution = 0.19 × 0.21 × 1 mm^3^, flip angle = 90°, and 5 *b*-value = 0, followed by 30 diffusion encoding gradient directions with a diffusion weighting of 660 s/mm^2^.

### Imaging data processing

All Bruker raw data were converted to Neuroimaging Informatics Technology Initiative (NIfTi) format using MRIcron V1 software (https://people.cas.sc.edu/rorden/mricron/).

#### Anatomical magnetic resonance imaging data analysis

T2-weighted images of the brain were preprocessed using the VBM tool VBM8 (Jena University Hospital, Germany; http://dbm.neuro.uni-jena.de/vbm/) of the SPM8 software package (UCL Queen Square Institute of Neurology, London, UK; https://www.fil.ion.ucl.ac.uk/spm). The first step was to define image orientation of the imaging data. According to the common three-dimensional coordinate system with three planes, we decided to implement the LIP system. Then, we used AFNI software version 23.1.10 (https://afni.nimh.nih.gov/) for the brain extraction. The resolution of mice MRI data is relatively high compared with human MRI data. To allow SPM to process the data, the data dimension had to be scaled by a factor of 10 to simulate human-similar voxel sizes. In this step, up- and down-scaling was carried out automatically only for the related NIfTi header file, whereas the voxel size of the raw image remained the same. This process was conducted by referring to the analysis methods used in previous literature on animal brain imaging (Pallast et al., 2019). Next, the scaled images were inputted to the SPM software for normalization. The registration procedure exclusively served the purpose of transferring structural data to the MSA mouse template in SPMmouse (https://www.fil.ion.ucl.ac.uk/spm/ext/#SPMMouse). Then, the normalized T2-weighted images were segmented into gray matter (GM), WM, and cerebrospinal fluid. The images needed to be roughly aligned with the mouse tissue probability maps, which was provided by SPMMouse. The segmented GM and WM images were then modulated to create a modified template using Diffeomorphic Anatomical Registration Through Exponentiated Lie Algebra (DARTEL) techniques based on the MSA mouse template. Flow fields were created for each mouse to store the Jacobian deformation information. The same fields were used to warp the segmented GM and WM to achieve good normalization. The final GM and WM images were smoothed with a 4-mm full-width at half maximum isotropic Gaussian kernel. Finally, the observed GM and WM images (resampled to 0.7 × 0.7 × 0.7 mm^3^) were analyzed using the SPM8 statistical software package to compare the volume differences between groups.

#### Diffusion magnetic resonance imaging data analysis

The DTI data were primarily processed using FMRIB software library version 6.0.2 (FSL, created by the Analysis Group, Oxford, UK). First, DTI data were converted to NIfTi format. The non-brain tissue was discarded by applying a binary mask of the brain extraction to the original DTI data. Then, to allow FSL to process the data, the data dimension needed to be scaled by a factor of 10 to simulate human-similar voxel sizes. DTI post-processing was performed using FSL software. FSL’s eddy correct tool was used to correct for distortions and motion artifacts (Graham et al., 2016). Tensors were fitted using the *b*-factor and diffusion direction matrix with the DTIfit toolbox. Eigenvalues (λ1, λ2 and λ3), the resulting eigenvectors and the FA indices were calculated for each voxel resulting in diffusion-weighted brain maps, including whole brain FA maps. Mean diffusion (MD) was defined as the mean of all three eigenvalues ((λ1 + λ2 + λ3)/3), RD as the mean of the second and third eigenvalues ((λ1 + λ2)/2), and AxD as the principal diffusion eigenvalue (λ1). The b0 images of each mouse were coregistered to a customized reference brain template (DARTEL template of T2-weighted data), and the resulting transforming matrix was then applied to register the respective DTI index maps. The normalized diffusion index maps (resampled to 0.7 × 0.7 × 0.7 mm^3^) were smoothed using an isotropic Gaussian filter (4-mm full-width at half maximum).

### Hippocampus tissue collection

After anesthetizing the animals as described above and determining deep anesthesia had been achieved by confirming the absence of reflexes, euthanasia was performed via cervical dislocation in a humane fashion. Immediately after death, hippocampal tissue samples of all mice were collected, flash-frozen in liquid nitrogen, and stored at –80°C.

### Immunofluorescence staining

Three mice in each group were anesthetized and then transcardially perfused with phosphate-buffered saline and 4% paraformaldehyde. After perfusion, the brains were carefully removed and fixed in 4% paraformaldehyde at 4°C overnight. Coronal brain sections of 20-μm thickness were used for immunofluorescence analysis. Sections were first permeabilized with 0.3% Triton X-100 in phosphate-buffered saline for 30 minutes at room temperature and then blocked with 3% bovine serum albumin for 1.5 hours at room temperature. Sections were then incubated with primary antibodies: mouse anti-Aβ clone 6E10 (1:1000, Cat# 803001, Biolegend, Dedham, MA, USA) and rabbit anti-phospho-Tau (Ser404) (1:1000, Cat# 2019s, CST, Danvers, MA, USA) at 4°C overnight. After primary antibody incubation, sections were washed and incubated for 2 hours at room temperature in the dark with corresponding secondary antibodies including goat anti-mouse Alexa Fluor 488 (1:1000, Cat# ab150113, Abcam, Cambridge, MA, USA) and goat anti-rabbit Alexa Fluor 488 (1:1000, Cat# ab150077, Abcam). Finally, the brain slices were mounted with 4,6-diamidino-2-phenylindole (DAPI) Fluoromount-G (Cat# 17985-50, EMS, Hatfield, PA, USA). Images were acquired using a laser scanning confocal microscope (Stellaris 5; Leica Microsystems, Berlin, Germany) and analyzed using ImageJ software (version 1.46r National Institutes of Health, Bethesda, MD, USA).

### Bulk RNA sequencing

The hippocampal tissues of three mice in the AD group and three in the control group underwent RNA sequencing. The total RNA from hippocampus tissue was extracted using TRIzol reagent (Accurate Biology, Changsha, China). Subsequently, the RNA samples were submitted to the Novogene Bioinformatics Institute (Beijing, China) for analysis. RNA integrity was assessed using the Bioanalyzer 2100 system with the RNA Nano 6000 Assay Kit (Agilent Technologies, Palo Alto, CA, USA). For cDNA library preparation, total RNA was purified following the manufacturer’s instructions using the AMPure XP system (Beckman Coulter, Beverly, MA, USA). The libraries were sequenced on an Illumina NovaSeq platform generating 150 bp paired-end reads for each sample. Differential expression analysis between the 3×Tg-AD group and control group was performed using DESeq2 R package version 1.20.0 (https://bioconductor.org/packages/release/bioc/html/DESeq2.html); a *P*-value threshold < 0.05 was considered significant for differentially expressed genes (DEGs). Visualization of DEGs was achieved through volcano plot and heatmap plot analyses using SangerBox (http://sangerbox.com/home.html). Additionally, Kyoto Encyclopedia of Genes and Genomes (KEGG) analysis was conducted using the KOBAS 2.0 database (http://bioinfo.org/kobas/annotate/) to identify significantly enriched metabolic pathways compared with the entire genome background.

### Statistical analyses

Sample sizes were not predetermined using statistical methods; however, our sample sizes are similar to those reported in previous publications (Manno et al., 2019; Rollins et al., 2019; Güell-Bosch et al., 2020). Data are presented as means ± standard deviation. Behavioral testing data were compared using the two-sample *t*-test (SPSS software 20.0; IBM Corp., Armonk, NY, USA).

For both the smoothed GM and WM maps, a two-tailed, two-sample *t*-test was conducted using linear model in SPM to compare brain volume information between the control and AD groups. The threshold for statistical significance of GM and WM volume changes was set at *P* < 0.05 with family-wise error correction applied. The mapping and naming of these different brain regions mainly referred to the Allen Mouse Brain Atlas (http://mouse.brain-map.org/static/atlas). Regions that survived this correction were considered significantly different and selected as ROIs. The GM and WM volumes of each ROI were extracted and listed in a histogram to display detailed changes in these regions.

Spatial-based voxel-wise statistics were also performed on the smoothed FA maps using two-tailed, two-sample *t*-tests to assess microstructural changes between the control and AD groups. The threshold for statistical significance of the FA maps was set at *P* < 0.005. The mapping and naming of these significant results mainly referred to the Allen Mouse Brain Atlas. Regions showing significant differences at this threshold were selected as ROIs. Mean FA values within each ROI were extracted and listed in a histogram for all mice included in the study. To explore the microstructural mechanisms underlying observed FA changes, diffusivity indices (AxD, MD, and RD) within ROIs exhibiting abnormal FA values were also investigated. Mean values of diffusion indices within these ROIs were calculated, followed by performing two-sample *t*-tests on average diffusivity indices between the two groups. A significance level of *P* < 0.005 was considered significant for average diffusivity index comparison.

Based on the aforementioned neuroimaging analyses, the regions exhibiting significant differences between the two groups were selected. The correlations between neuroimaging parameters (volume and diffusion indices) and behavioral performances (open field test, Y-maze test, and MWM test) were assessed using Pearson’s method. *P* < 0.05 was considered significant.

## Results

### Behavioral impairment in the 3×Tg-AD mice

**Figure 1:**
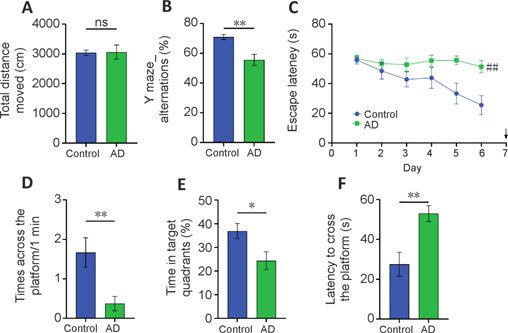
Assessment results of the open field test, Y maze test, and Morris water maze test. (A) Total distance traveled in the open field test. (B) Y maze alternation. (C) Escape latency during the place navigation test, (D) the number of platform crossings, (E) the time spent in the target quadrant, (F) and the escape latency in the probe trial in the Morris water maze test. All data are presented as the mean ± SEM (*n* = 9 in each group). *P < 0.05, **P < 0.01. ##*P* < 0.01, *vs*. control group.

To investigate cognitive behavioral impairments such as movement and memory in AD animals at the age of 9 months, the following behavioral experiments were conducted. The open field test assessed locomotor activity, revealing no significant differences in distance traveled or speed between the 3×Tg-AD mice and controls (**Figure 1A**). In the Y-maze test, spontaneous alternation was significantly lower in the 3×Tg-AD mice (55.5% *vs.*70.9%; *P* < 0.01; **Figure 1B**). In the MWM test, the 3×Tg-AD mice took significantly longer to locate the invisible platform on the sixth day of training (51.3 seconds *vs*. 25.4 seconds; *P* < 0.01; **Figure 1C**). Furthermore, they crossed the platform significantly less frequently (0.4 crossings *vs*. 1.7 crossings; *P* < 0.01; **Figure 1D**), spent a lower percentage of time in the target quadrant (24.3% *vs*. 36.9%; *P* < 0.05; **Figure 1E**) and had a higher latency for crossing the platform (52.9 seconds *vs*. 27.5 seconds; *P* < 0.01; **Figure 1F**). The results of the behavioral experiments showed that the cognitive abilities such as movement and memory of the AD mice at this age had already been impaired.

### Voxel-based morphometry analysis of gray matter and white matter volumes

**Figure 2:**
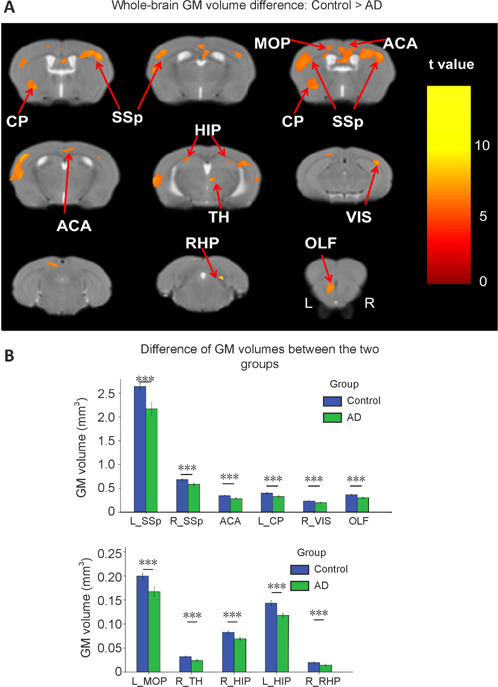
Main results of the whole-brain GM volume differences between the two groups according to voxel-based morphometry analysis of the T2-weighted images. (A) The GM volume in the 3×Tg-AD group was markedly lower than that in the control group. (B) The regions showing significant differences were selected as regions of interest, and the GM volumes in these regions. All data are presented as the mean ± SEM (*n* = 9 in each group). ***P < 0.001. ACA: Anterior cingulate; CP: caudoputamen; GM: Gray matter; HIP: hippocampal region; L: left; MOP: primary motor area; OLF: olfactory bulb; R: right; RHP: retrohippocampal region; SSP: primary somatosensory area; TH: thalamus; VIS: retrolateral lateral visual area.

To demonstrate whether there was any damage to brain structure in 9-month-old AD mice, the GM volumes were calculated of the whole brain. The GM results from VBM analysis are presented in **Figure 2A**. Compared with the control group, the 3×Tg-AD group exhibited a significant decrease in GM volume, primarily in the bilateral hippocampi, right retrohippocampal region, right subparafascicular area of thalamus, bilateral primary somatosensory area, left caudoputamen (CP), left primary motor area, right anterolateral visual area, anterior cingulate area, and olfactory bulb (OLF). These regions displaying abnormal GM volume were selected as ROIs and their respective GM volume values were extracted for all mice. The altered GM volume information within these regions was compiled into a histogram, as shown in **Figure 2B** (*P* < 0.05 with family-wise error correction).

**Figure 3:**
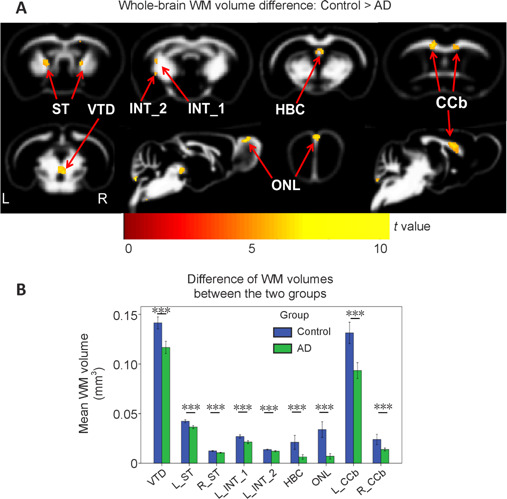
Main results of the whole-brain WM volume differences between the two groups according to voxel-based morphometry analysis of the T2-weighted images. (A) Regions showing a significant decrease in white matter volume between the two groups. (B) The regions showing significant differences were selected as regions of interest, and the WM volumes in these regions are listed in the histogram. All data are presented as the mean ± SEM (*n* = 9 in each group). ***P < 0.001 (two-sample *t*-test). CCb: Body of the corpus callosum; HBC: habenular commissure; INT: internal capsule; L: left; ONL: olfactory nerve layer of the main olfactory bulb; R: right; ST: stria terminalis; VTD: ventral tegmental decussation.

To better demonstrate the location of the volume difference in the WM, we overlaid the WM results on the WM probability atlas from SPMmouse software. The WM results from the VBM analysis are presented in **Figure 3A**. Compared with the control group, the 3×Tg-AD group exhibited a significantly lower WM volume, which was primarily localized in multiple fiber tracts, including the body of the corpus callosum (CCb), bilateral stria terminalis, left internal capsule, habenular commissure, olfactory nerve layer of the main OLF (ONL), and ventral tegmental decussation. These regions displaying abnormal WM volume were selected as ROIs and the corresponding WM volume values were extracted for all mice. The altered information regarding WM volume in these regions was compiled into a histogram, as depicted in **Figure 3B** (*P* < 0.05 with family-wise error correction).

### Voxel-based diffusion tensor imaging alterations

**Figure 4:**
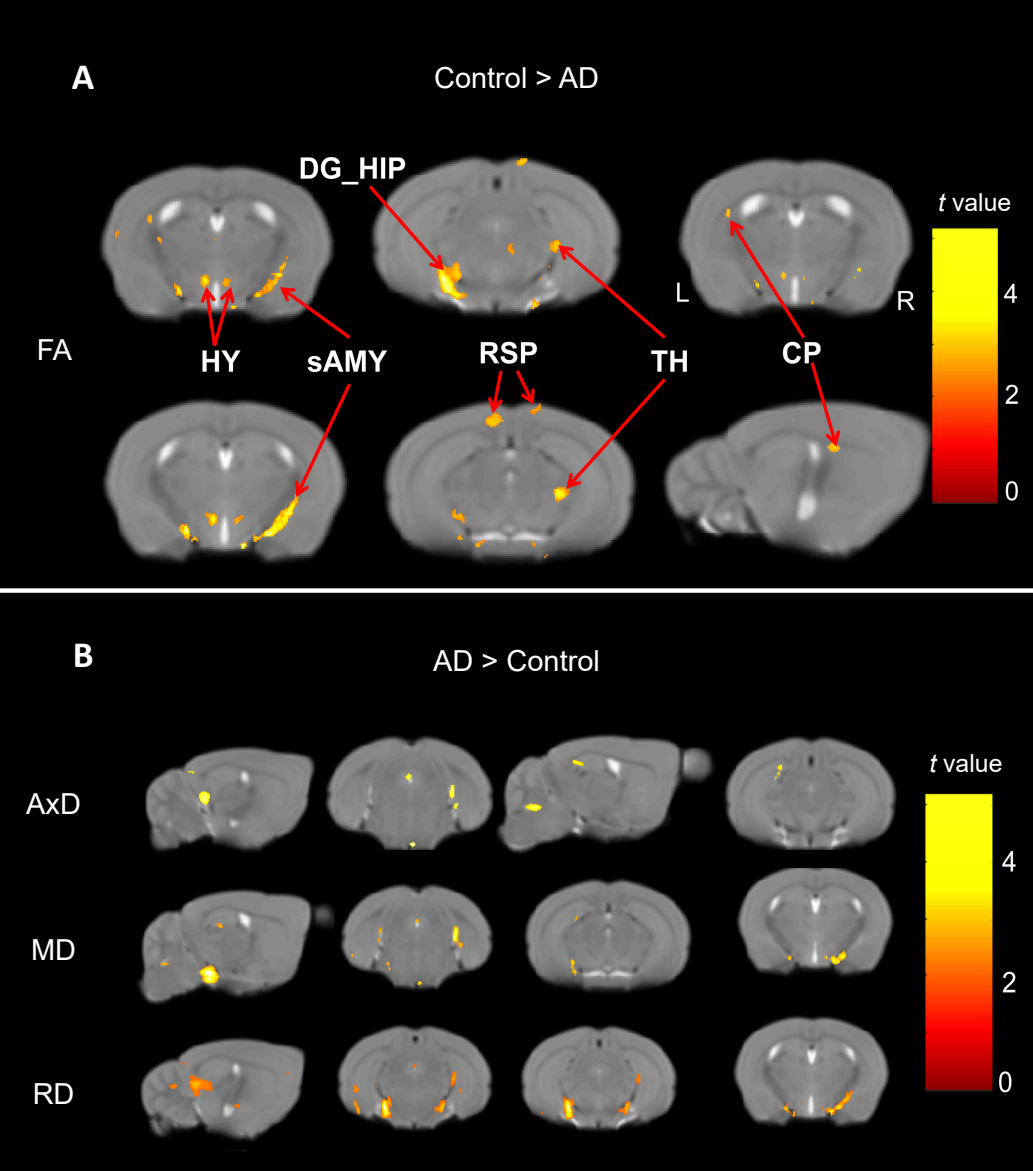
FA diffusion maps revealing WM microstructural differences between the two groups. (A) Untreated 3×Tg-AD model mice presented a significant decrease in FA compared with control mice according to voxel-based statistics. (B) The regions showed significant decrease of the diffusion index (AxD, MD, and RD) in the 3×Tg-AD group compared with the control group. CP: Caudoputamen; DG_HIP: dentate gyrus of the hippocampal region; HY: hypothalamus; L: left; R: right; RSP: retrosplenial area; sAMY: striatum-like amygdala nuclei; TH: thalamus.

To examine microstructural changes in AD mice, the diffusion of water molecules throughout the whole brain was measured using DTI. The results of the DTI analysis are presented in **Figure 4**. **Figure 4A** displays marked clusters indicating lower FA in the 3×Tg-AD group than the control group, which was primarily observed in the bilateral HY, bilateral retrosplenial area (RSP), left CP, left dentate gyrus of the hippocampal region (DG_HIP), right dorsal TH, and striatum-like amygdala nuclei (sAMY) (*P* < 0.005). **Figure 4B** illustrates marked clusters obtained through whole brain voxel-based analysis, revealing higher diffusion indices (AxD, MD, and RD) in the 3×Tg-AD group than the control group. In terms of MD and RD diffusion maps, clusters were predominantly found in the left DG_HIP and bilateral sAMY.

**Figure 5:**
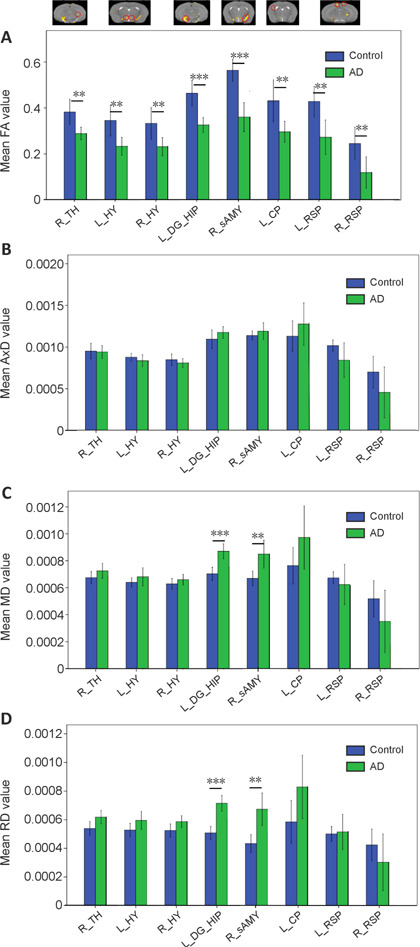
Bar plots showing the averaged diffusion tensor metrics. The regions showing a significant decrease in FA in Figure 4A were selected as regions of interest, and the averaged diffusion tensor metrics (FA, AxD, MD, and RD) were selected and compared between groups. (A) Quantitative analysis of the FA. (B) Quantitative analysis of AxD. (C) Quantitative analysis of the MD. (D) Quantitative analysis of the RD. All data are presented as the mean ± SEM (*n* = 9 in each group). **P < 0.01, ***P < 0.001 (two-sample *t*-test). CP: Caudoputamen; DG_HIP: dentate gyrus of the hippocampal region; FA: fractional anisotropy; HY: hypothalamus; RSP: retrosplenial area; sAMY: striatum-like amygdala nuclei; TH: thalamus.

The regions exhibiting abnormal FA values were selected as ROIs for further analysis where mean diffusion indices values were extracted from each ROI across all mice. Subsequently, these diffusion indices within these regions were compiled into a histogram as depicted in **Figure 5** (*P* < 0.005). Compared with the control group, the AD group exhibited significantly decreased mean FA values in all ROIs (**Figure 5A**). In contrast, the 3×Tg-AD group showed no significant changes in AxD values in any of the DTI ROIs compared with controls (**Figure 5B**). Furthermore, the 3×Tg-AD group had significantly higher mean MD and RD values in the right sAMY and left DG_HIP than the control group (**Figure 5C** and **D**).

### Correlation results

Based on the GM VBM results mentioned above, we selected the regions that exhibited significant differences between the two groups as our areas of interest. The GM volumes in these regions were then examined for correlations with behavioral data. **Table 1** presents the correlation findings, indicating that all these areas showed significant and positive relationships between their GM volumes and Y-maze performance as well as time spent across the platform. Additionally, there were significant and negative relationships observed between the GM volumes of these areas and both escape latency from the invisible platform and latency across the platform.

**Table 1:** Correlations between brain anatomical volume and behavioral performance

Regions	Y maze			Escape latency			Time across the platform			Time in target quadrants			Latency to cross the platform	
								
	*r* value	*P*-value		*r* value	*P*-value		*r* value	*P*-value		*r* value	*P*-value		*r* value	*P*-value
Gray matter volumes														
L_SSR	0.528	0.024		–0.476	0.046		0.710	0.001		0.174	0.489		–0.783	0
R_SSR	0.527	0.025		–0.567	0.014		0.745	0		0.259	0.300		0.701	0.001
ACA	0.597	0.009		–0.588	0.010		0.681	0.002		0.196	0.436		–0.686	0.002
L_MOP	0.498	0.035		–0.587	0.010		0.719	0.001		0.067	0.792		–0.707	0.001
L_CP	0.528	0.024		–0.476	0.046		0.710	0.001		0.174	0.489		–0.783	0
R_TH	0.482	0.043		–0.576	0.012		0.644	0.004		0.188	0.456		–0.67	0.002
R_VIS	0.619	0.006		–0.562	0.015		0.655	0.003		0.196	0.436		–0.672	0.002
R_HIP	0.592	0.010		–0.467	0.050		0.527	0.025		0.189	0.453		–0.638	0.004
L_HIP	0.588	0.010		–0.504	0.033		0.642	0.004		0.039	0.879		–0.700	0.001
OLF	0.588	0.010		–0.667	0.003		0.651	0.003		0.151	0.550		–0.667	0.003
R_RHP	0.535	0.022		–0.626	0.005		0.520	0.027		0.236	0.345		–0.602	0.008
White matter volumes														
VTD	0.510	0.031		–0.402	0.098		0.439	0.038		0.311	0.209		–0.688	0.002
L_ST	0.437	0.070		–0.466	0.052		0.522	0.026		0.077	0.760		–0.721	0.001
R_ST	0.581	0.012		–0.469	0.050		0.498	0.035		0.296	0.233		–0.694	0.001
L_INT_1	0.314	0.205		–0.381	0.119		0.701	0.001		0.175	0.488		–0.635	0.005
L_INT_2	0.553	0.017		–0.490	0.039		0.498	0.035		0.066	0.794		–0.664	0.003
HBC	0.230	0.359		–0.234	0.350		0.741	0		0.255	0.307		–0.572	0.013
ONL	0.516	0.029		–0.442	0.066		0.564	0.015		0.166	0.512		–0.555	0.017
L_CCb	0.465	0.052		–0.537	0.022		0.798	0		0.084	0.740		–0.638	0.004
R_CCb	0.329	0.183		–0.299	0.228		0.671	0.002		0.045	0.858		–0.437	0.069

Significant results are reported in bold. ACA: Anterior cingulate area; CCb: body of the corpus callosum; CP: caudoputamen; HBC: habenular commissure; HIP: hippocampus; INT: internal capsule; L: left; MOP: primary motor area; OLF: olfactory bulb; ONL: olfactory nerve layer of the main olfactory bulb; R: right; RHP: retrohippocampal region; SSR: primary somatosensory area; ST: stria terminalis; TH: subparafaxicular area of the thalamus; VIS: anterolateral visual area; VTD: ventral tegmental decussation.

Similarly, based on the WM VBM results discussed earlier, we identified regions that displayed significant differences between the two groups as areas of interest. Subsequently, we investigated correlations between WM volumes in these regions and behavioral data. **Table 1** provides an overview of these correlation results, revealing that most of these areas (ventral tegmental decussation, right stria terminalis, left internal capsule, OLF and left CCb) demonstrated significant and positive relationships with Y-maze performance along with time spent across the platform. Furthermore, a majority of these areas exhibited significant negative relationships with both escape latency from the invisible platform and latency across it.

**Figure 6:**
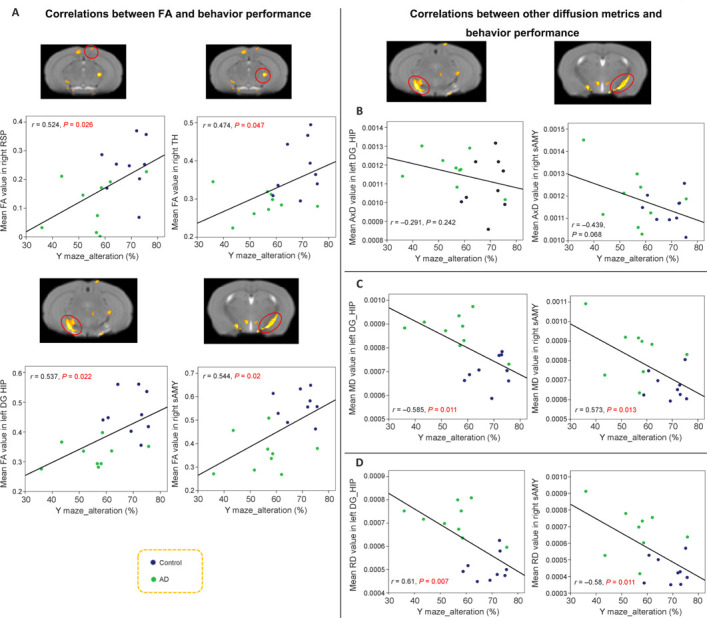
Correlational analysis between the diffusion index and behavioral performance. (A) Correspondence analysis between Y maze alterations and the FA values of regions of interest (left DG_HIP and right sAMY). (B) Correspondence analysis between the Y maze alteration and the AxD values of regions of interest (left DG_HIP and right sAMY). (C) Correspondence analysis between Y maze alterations and the MD values of regions of interest (left DG_HIP and right sAMY). (D) Correspondence analysis between Y maze alterations and RD values of regions of interest (left DG_HIP and right sAMY). AxD: Axial diffusion; DG_HIP: Dentate gyrus of the hippocampal region; FA: fractional anisotropy; MD: mean diffusion; sAMY: striatum-like amygdala.

Based on the results obtained from the DTI analysis of FA maps, we selected regions that exhibited a significant difference in FA between the two groups as areas of interest. The mean FA values in these regions were then examined for correlations with behavioral data. **Figure 6A** illustrates significantly positive correlations between Y-maze performance and the mean FA values in the right RSP, right thalamus, left DG_HIP, and right sAMY. Additionally, we extracted mean values of other diffusion metrics (such as AxD, MD, and RD) from these areas of interest. Correlation analyses further revealed significant negative associations between Y-maze performances and the mean MD and RD values in the left DG_HIP and right sAMY (**Figure 6B–D**). We also calculated relationships between diffusion indices and MWM test results. Significant correlations were primarily observed in the left DG_HIP and right sAMY. Detailed information regarding these findings can be found in **Additional Table 1**.

**Additional Table 1:** Correlations between brain diffusion indices and behavioral performance

Image indices	Regions	Y maze		Escape latency		Time across the platform		Time in target quadrants		Latency to cross the platform	

		*r* value	*P*-value	*r* value	*P*-value	*r* value	*P*-value	*r* value	*P*-value	*r* value	*P*-value
**FA**	L_HY	0.283	0.255	**-0.482**	**0.043**	**0.662**	**0.003**	0.243	0.332	-0.357	0.146
	R_HY	0.322	0.192	-0.395	0.105	**0.674**	**0.002**	0.229	0.361	-0.374	0.126
	L_RSP	0.372	0.128	**-0.477**	**0.045**	**0.840**	**0.000**	0.157	0.534	**-0.632**	**0.005**
	R_RSP	**0.524**	**0.026**	-0.426	0.078	**0.479**	**0.044**	0.106	0.674	**-0.505**	**0.033**
	R_TH	**0.474**	**0.047**	**-0.628**	**0.005**	0.464	0.053	0.429	0.076	-0.419	0.084
	L_DG_HIP	**0.537**	**0.022**	**-0.503**	**0.034**	**0.577**	**0.012**	0.144	0.569	-0.414	0.087
	R_AMY	**0.544**	**0.020**	**-0.563**	**0.015**	**0.626**	**0.005**	0.229	0.362	**-0.843**	**0.000**
	L_CP	0.151	0.551	-0.163	0.518	0.402	0.098	0.087	0.732	-0.276	0.268
**AxD**	L_HY	**0.547**	**0.019**	-0.305	0.219	0.231	0.356	0.169	0.503	-0.140	0.579
	R_HY	0.428	0.076	-0.126	0.617	0.035	0.890	0.118	0.642	-0.150	0.553
	L_RSP	0.34	0.168	-0.254	0.309	0.460	0.055	0.120	0.635	-0.364	0.138
	R_RSP	0.440	0.068	-0.200	0.429	0.264	0.290	0.058	0.818	-0.338	0.170
	R_TH	0.327	0.185	-0.220	0.380	0.021	0.934	0.309	0.212	0.035	0.891
	L_DG_HIP	-0.291	0.242	0.183	0.466	-0.203	0.420	-0.150	0.554	0.395	0.104
	R_AMY	-0.439	0.068	0.281	0.259	-0.408	0.093	0.422	0.093	0.420	0.083
	L_CP	0.016	0.950	0.271	0.276	-0.439	0.068	-0.439	0.068	0.413	0.088
**MD**	L_HY	0.216	0.389	0.130	0.607	-0.241	0.336	0.059	0.815	0.183	0.746
	R_HY	0.105	0.678	0.298	0.230	-0.418	0.084	-0.082	0.746	0.197	0.434
	L_RSP	0.175	0.488	-0.004	0.988	0.120	0.635	0.117	0.644	-0.170	0.501
	R_RSP	0.402	0.098	-0.137	0.589	0.221	0.378	0.050	0.842	-0.312	0.208
	R_TH	0.068	0.789	0.184	0.466	-0.294	0.237	0.048	0.850	0.336	0.173
	L_DG_HIP	**-0.585**	**0.011**	**0.492**	**0.038**	**-0.588**	**0.010**	-0.210	0.402	**0.637**	**0.004**
	R_AMY	**-0.573**	**0.013**	**0.492**	**0.038**	**-0.608**	**0.007**	0.089	0.725	**0.763**	**0.000**
	L_CP	-0.121	0.632	0.293	0.239	-0.458	0.056	-0.109	0.668	0.409	0.092
**RD**	L_HY	0.040	0.874	0.292	0.239	-0.380	0.120	0.034	0.893	0.228	0.362
	R HY	-0.101	0.690	0.401	0.099	**-0.529**	**0.024**	-0.111	0.661	0.315	0.203
	L_RSP	0.009	0.910	0.214	0.394	-0.183	0.467	0.097	0.702	0.021	0.934
	R_RSP	0.353	0.151	0.054	0.833	0.165	0.513	0.018	0.943	-0.265	0.288
	R_TH	-0.160	0.526	0.435	0.071	-0.439	0.069	-0.130	0.606	0.444	0.065
	L_DG_HIP	**-0.610**	**0.007**	**0.550**	**0.018**	**-0.650**	**0.004**	-0.178	0.480	**0.613**	**0.007**
	R_AMY	**-0.581**	**0.011**	**0.533**	**0.023**	**-0.615**	**0.007**	-0.017	0.947	**0.805**	**0.000**
	L_CP	-0.174	0.489	0.286	0.250	-0.410	0.091	-0.115	0.651	0.349	0.156

Significant results are reported in bold type. AMY: Amygdala nuclei; AxD: axial diffusion; CP: caudoputamen; DG HIP: dentate gyrus of the hippocampus; FA: fractional anisotropy; HY: amygdala nuclei; L: left; MD: mean diffusion; R: right; RD: radial diffusion; RSP: retrosplenial area; TH: subparafaxicular area of the thalamus.

### Pathology of amyloid-β and tau in 3×Tg-AD mice

**Figure 7:**
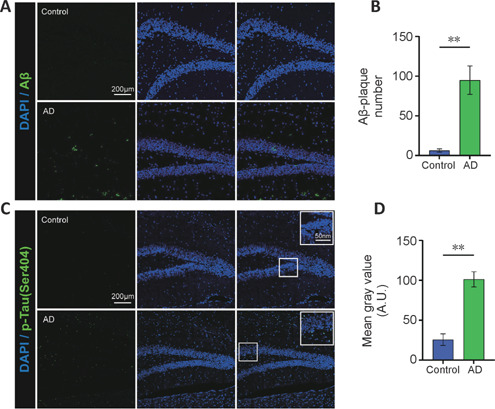
Pathology of Aβ and tau in 3×Tg-AD mice. (A) Representative images of Aβ (green) and DAPI (blue) immunofluorescence staining in the hippocampus. (B) Quantification of the number of Aβ plaques (*n* = 3 in each group). (C) Representative immunofluorescence images of p-Tau (Ser404) (green) with DAPI (blue) in the hippocampus. (D) Quantification of the mean gray value of p-Tau (Ser404) (*n* = 3 in each group). Scale bars: 200 μm/50 nm in A and C. All the data are presented as the mean ± SEM. **P < 0.01 (two-sample *t*-test). Aβ: Amyloid-β; DAPI: 4′,6-diamidino-2-phenylindole.

To further confirm their relationship with neuroimaging and gene expression findings, immunofluorescence analysis of Aβ and p-Tau (Ser404) in coronal brain sections was performed. The results demonstrated that the number of Aβ plaques in the hippocampus was significantly higher in the AD group than the controls (*P* < 0.01; **Figure 7A** and **B**); this was accompanied by an upregulation in p-Tau (Ser404) expression level (*P* < 0.01; **Figure 7C** and **D**).

### RNA sequencing data: Differential expression and pathway analysis results

**Figure 8:**
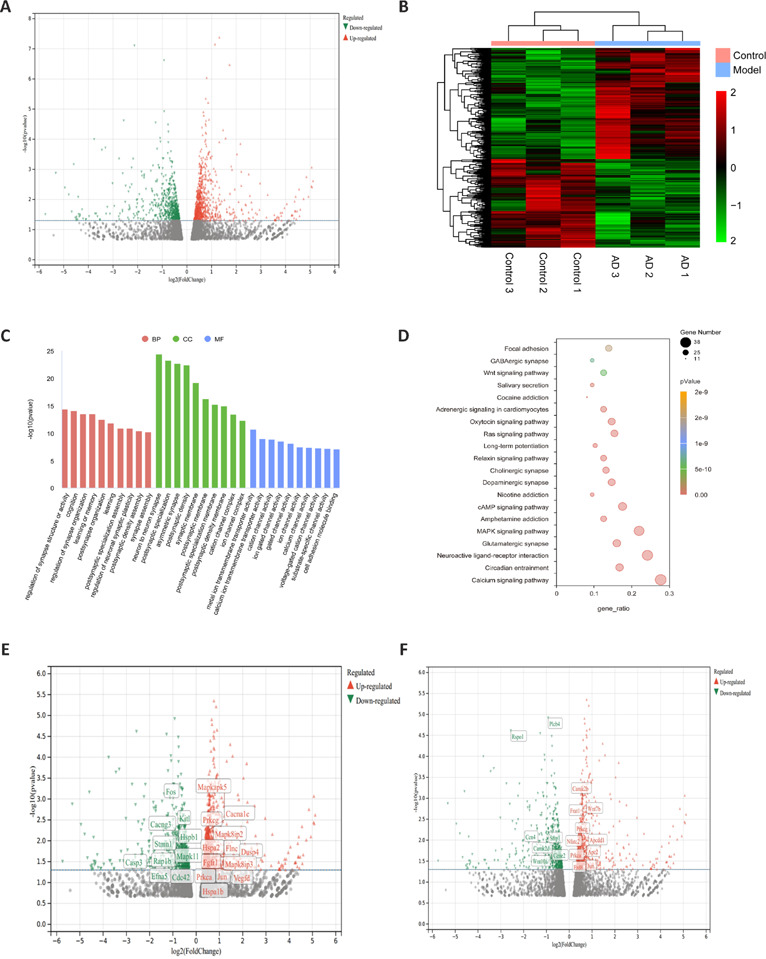
Bulk RNA sequencing results between the model and control groups. (A) Volcano plot and (B) heatmap plot of DEGs. (C) Gene Ontology enrichment analysis. (D) Kyoto Encyclopedia of Genes and Genomes pathway analysis. (E) Significant DEGs in the MAPK signaling pathway. (F) Significant DEGs in the Wnt signaling pathway. BP: Biological process category; CC: cellular component category; DEGs: differentially expressed genes; MF: molecular function category.

Bulk RNA-seq was conducted to further investigate the alterations in the 3×Tg-AD mice. The findings revealed differential expression of 1389 genes between the two groups, with 774 genes up-regulated and 615 genes down-regulated in the 3×Tg-AD group (**Figure 8A** and **B**). The significant difference in gene expression is valuable for studying the molecular mechanisms underlying structural differences in the hippocampus region between AD and control mice.

Gene Ontology (GO) enrichment analysis was performed on DEGs, resulting in significant enrichment of 10 GO terms each within biological process, molecular function, and cellular component categories. Importantly, many of these significantly enriched GO terms are closely associated with cognition and memory. As shown in **Figure 8C**, numerous genes within the biological process category are enriched in synapse structure and function, cognition, learning, and memory. These GO terms indicate that DEGs might play important roles in structural changes in the hippocampus.

KEGG pathway analysis revealed that these significantly enriched genes were involved in 20 signaling pathways according to the KEGG database. **Figure 8D** illustrates some pathway enrichment results related to nerve myelination processes, including the Wnt signaling pathway and mitogen-activated protein kinase (MAPK) signaling pathway. **Figure 8E** demonstrates that among the 23 *DEGs*, *Fos*, *Casp3*, *Mapk11*, *Mapkapk5*, *Mapk8ip2*, and *Jun* are associated with the MAPK signaling pathway. Furthermore, **Figure 8F** showed that among the 17 DEGs (such as *Rspo1*, *Plcb4*, *Wnt10a*, *Wnt7b*, and *Fzd8*), there is an association with the Wnt signaling pathway. **Additional Table 1** provides detailed information about DEGs obtained from GO enrichment analysis.

## Discussion

In the present study, we combined VBM of T2-weighted MRI with whole-brain voxel-based analyses of DTI to investigate changes in brain volume and microstructure between 9-month-old 3×Tg-AD mice and normal controls. Brain volumes were assessed using VBM, which revealed significantly lower GM volumes in the bilateral hippocampi, right thalamus, left CP, left OLF, and cortex (bilateral primary somatosensory area, left primary motor area, right anterior cingulate, and anterolateral visual area) in the 3×Tg-AD mice. The WM fiber tracts connecting these GM regions also showed significantly lower volume (CCb, internal capsule, habenular commissure, stria terminalis, ventral tegmental decussation, and ONL). Whole-brain DTI analyses detected significant decreases in FA in the bilateral HY, right thalamus, left dentate gyrus, right sAMY, left CP, and cortex (bilateral RSP). Additionally, significant enhancements of MD and radial diffusivity values were observed in the left dentate gyrus and right sAMY within the AD group. To determine neuroimaging biomarkers, we selected the above indices from VBM and DTI analysis results that showed significant correlations with behavioral performance across most interesting regions, particularly within the left hippocampus and right sAMY. To comprehend the neuroimaging alterations, we further conducted RNA-seq analysis to compare the gene expression profiles in the hippocampal region between the AD and control mice. Subsequent GO and KEGG pathway analyses unveiled that numerous DEGs were significantly enriched in synapse structure, learning and memory processes, as well as pathways associated with nerve myelination processes. These findings demonstrate widespread anatomical and microstructural changes occurring at a stage characterized by extensive Aβ deposition and onset of tau pathology in 3×Tg-AD mice. The RNA-seq results obtained from the hippocampus region may suggest that variations in gene expression could potentially contribute to the observed neuroimaging changes. Mice exhibiting lower Y-maze behavioral performance as well as longer time spent crossing the platform exhibited significant alterations in their brain neuroimaging indices. These results provide insights into neural mechanisms during this specific stage of pathology transformation in the 3×Tg-AD mouse model.

### Gray matter volume differences between the 3×Tg-AD and control groups

AD is associated with progressive cerebral atrophy. Previous neuroimaging studies comparing GM changes between AD patients and healthy individuals have identified significant morphological atrophy in the hippocampus, entorhinal cortex, amygdala, putamen, and thalamus (de Jong et al., 2008; Serra et al., 2010; Matsuda, 2013). At the preclinical stage of AD, parahippocampal gyrus atrophy has also been detected in patients with mild cognitive impairment (Huang et al., 2023). Consistent findings regarding brain neuroimaging changes have emerged from these previous AD studies, which indicate that patients at different stages of AD exhibit specific structural changes corresponding to the specific pathological changes of each stage. This perspective has also been supported by preclinical mouse model studies. Longitudinal studies have revealed significant changes in brain volumes (both increases and decreases) in 3×Tg-AD mice during the early stages from 1.5 months to 6 months of age (Kong et al., 2018; Rollins et al., 2019). Another longitudinal study using the same mouse model found a significant decrease in the hippocampus, cerebellum, OLF and cortex from 5 to 12 months (Güell-Bosch et al., 2020). These studies suggest that neuroanatomical alterations are observed prior to the manifestation of AD-like behaviors.

Notably, we found that 3×Tg-AD mice had significantly lower GM volumes in the bilateral hippocampi, bilateral thalami, left CP, OLF and cortex (bilateral primary somatosensory area, left primary motor area, right anterior cingulate and anterolateral visual area) at the age of 9 months. The morphological changes observed in the hippocampus, OLF, and cortex in our study were consistent with those in previous 3×Tg-AD mouse model studies (Liu et al., 2018; Rollins et al., 2019; Güell-Bosch et al., 2020). Because brain atrophy in the hippocampus, OLF, and cortex have been detected in 3×Tg-AD mice before age 9 months, persistent brain atrophy in these regions can be easy to understand. As the disease progresses from 2 to 9 months, amyloid deposits begin to propagate from the hippocampus to other regions, accompanied by the emergence of tau pathology. Low GM volumes in the thalamus and CP of 9-months-old 3×Tg-AD mice may reflect pathological changes captured through neuroimaging. Therefore, our findings align with previously published research regarding spatial patterns of decreased GM volume. Specifically, alterations in the bilateral thalami and left CP can be considered as distinct neuroimaging outcomes specific to 3xTg AD mice at 9 months.

### White matter volume differences between the 3×Tg-AD and control groups

Another main finding is that 3×Tg-AD mice exhibited significantly lower brain WM volumes, specifically in the fiber tracts of the bilateral CCb, bilateral stria terminalis, left internal capsule, habenular commissure, and OFL. VBM analysis of GM volume is widely used in patients with AD (Matsuda, 2013; Huang et al., 2023) and preclinical animal models (Liang et al., 2017; Bergamino et al., 2023; Li, 2024). Although VBM analysis of WM has been employed in previous clinical studies of patients with AD (Serra et al., 2010; Li et al., 2012), our study represents the first application of VBM to investigate WM changes in 3×Tg-AD mice. We identified WM atrophy in these mice, which aligns with the observed trends from previous clinical studies.

When we examined specific brain regions that exhibited WM changes, we found that these regions were also reported in previous preclinical models of AD (Zerbi et al., 2013; Kong et al., 2018; Praet et al., 2018; Falangola et al., 2020; Güell-Bosch et al., 2020; Jullienne et al., 2022). The CC is the major collection of WM bundles connecting both hemispheres, playing key roles in integration of perception and action. The internal capsule carries information past the basal ganglia and transmits signals from the primary motor cortex to the lower motor neurons of the spinal cord. Previous studies on 3×Tg-AD mice have reported significant volumetric decreases (Kong et al., 2018) and WM microstructural abnormalities (Praet et al., 2018; Gatto et al., 2019; Bergamino et al., 2023) in the CC and internal capsule compared with controls. Our results align with these studies, indicating that WM injury in AD may primarily result from axon loss and myelin breakdown.

The OLF is affected early in AD, playing an important role in the onset and progression of the disease (Zhou et al., 2024). A previous neuroimaging study showed that olfactory impairment is associated with hippocampal atrophy and entorhinal cortical thinning in preclinical AD (Hagan, 2023). Several preclinical studies have also detected structural changes in the olfactory system of AD mice (Kong et al., 2018; Chiquita et al., 2019). The pattern of early and consistent involvement of the olfactory system in AD, as indicated by previous studies, suggests that olfactory function might be particularly vulnerable and could reflect the disease process (Li et al., 2012; Zhou et al., 2024). In our study, we found that WM volumes in the ONL were significantly lower in the AD group. This result is consistent with those in previous studies, and reflects the vulnerability of the olfactory system to AD. The stria terminalis is a major efferent tract of the amygdala, with fibers projecting to the anterior HY. A previous study in a mouse model of AD reported lower volumes in the right stria terminalis at 6 months of age (Rollins et al., 2019). Our results demonstrated significantly lower WM volumes in the bilateral stria terminalis. The discrepancies between our results and those in previous studies may arise from the fact that our mice were 9 months old, whereas previous ones focused on younger mice, suggesting that WM fiber disruption in the stria terminalis progresses from one hemisphere to both as the disease progresses.

To better explain the results of WM volume change, we need to combine these findings with those of GM VBM analysis. Brain atrophy is a prominent imaging characteristic of AD. Lower GM volumes in the bilateral hippocampi, CP, thalamus, and cortex were commonly observed in our GM VBM results and in prior studies (Maheswaran et al., 2009; Pini et al., 2016; Jullienne et al., 2022). The structural abnormalities in these GM regions may result from damage to the nerve fiber connections within them. Our VBM results for WM support this perspective, indicating that fiber tract volumes in the CC, stria terminalis, internal capsule, and ONL were significantly lower in the AD group. These findings suggest that abnormal structural pathways in AD may be the combined outcome of axon loss or myelin breakdown (Dong et al., 2018). By integrating the findings from our GM and WM analyses, we observed localized brain atrophy expanding from GM areas into WM regions of 9-month-old 3×Tg-AD mice.

Another approach to interpret the WM VBM results is to consider the behavioral data. The fiber tracts exhibiting a significantly lower volume are all involved in high-order cognitive functions. The behavioral experiment conducted in this study revealed significantly worse Y-maze performance in 9-month-old 3×Tg-AD mice compared with age-matched controls. This finding suggests that working memory ability begins to deteriorate at this age in these mice. The behavioral results partially support the WM VBM findings, leading us to infer that the observed WM atrophy may indicate disruption in effective connectivity within the brain, although further verification is required.

### Diffusion tensor imaging changes in the 3×Tg-AD mouse model

Microstructural differences in WM between the 3×Tg-AD mice and controls were also assessed. Previous DTI studies have demonstrated WM integrity impairment in various types of AD mouse models (Qin et al., 2013; Massalimova et al., 2020; Bergamino et al., 2023). Most reported DTI abnormalities characterized by reduced FA and both decreased/increased RD in the AD group. However, when focusing specifically on 3×Tg-AD model mice, previous DTI investigations yielded inconsistent results. One study involving mice aged between 11 and 17 months found no WM DTI changes compared with controls (Kastyak-Ibrahim et al., 2013). Conversely, other studies conducted on the same model in mice at ages 2, 12, and 14 months identified significant WM alterations (Manno et al., 2019; Nie et al., 2019; Falangola et al., 2021). In our study, voxel-based analyses of DTI were employed to detect whole-brain diffusion changes in the brain of 3×Tg-AD mice. The model mice exhibited a significant decrease in FA and an increase in MD and RD at 9 months of age. The directionality of DTI metrics observed in our study is generally consistent with most previous findings and highlights that outcomes are dependent on both age and region location within the model. For instance, a prior investigation discovered decreased AxD and FA values within the basal forebrain of 2-month-old 3×Tg-AD mice, with progressive temporal declines in AxD and FA at 8 and 15 months of age (Falangola et al., 2021). Within the hippocampus, significant changes of diffusion metrics (FA, MD, AxD, and RD) were observed in 3×Tg-AD mice compared with controls, with decreased FA and AxD at both 2 and 8 months (Falangola et al., 2021), and increased RD at 12 and 14 months (Snow et al., 2017). Different regions exhibited distinct trajectories of change throughout the lifespan of AD mice. In this study, a decrease in FA, indicative of reduced WM integrity, was primarily observed in the bilateral HY, right thalamus, left dentate gyrus, right sAMY, left CP, and cortex (bilateral RSP). Conversely, increased MD and RD were mainly observed in the left dentate gyrus and right sAMY. One explanation for these diffusion metric changes in the hippocampus and sAMY is that GM degeneration leads to microstructural abnormality through demyelination. These results support the notion that myelin damage caused by AD pathology can be clearly detected using DTI methods, particularly evidenced by the well-established alterations in myelination patterns in 9-month old 3×Tg-AD mice. The reduced integrity observed at this stage may reflect specific pathology patterns characterized by extensive intracellular Aβ aggregates and emerging extracellular Aβ and tau immunoreactivity (Falangola et al., 2022). At this particular stage, myelin damage in the left dentate gyrus and right sAMY may represent key features of pathological transformation. When combined with behavioral results and voxel-based morphometry findings, it becomes apparent that there are anatomical and microstructural changes associated with AD pathology specifically within memory-related areas. Consequently, memory impairment occurs alongside depressive-like behavior exhibited by these mice. This interpretation aligns with neuroimaging-behavior correlation findings discussed later.

### Neuroimaging and behavior relationships

To investigate the neural mechanisms underlying the observed MRI findings, we further examined the relationship between the MRI metrics and behavioral performance. As anticipated, there were significant positive correlations between the MRI indices of the most intriguing regions and both Y-maze alterations and platform crossings. Additionally, these MRI indices exhibited significant negative correlations with escape latency from an invisible platform and latency across the platform in the MWM test. The Y-maze task is a widely used behavioral experiment in mouse models for assessing working memory. At 9 months of age, 3×Tg-AD mice displayed a significant decrease in Y-maze alteration. The regions showing significant correlations are primarily located within the hippocampus and sAMY, which are involved in memory formation processes. This study revealed that mice with lower Y-maze alteration exhibited reduced GM volume, decreased FA, increased MD, and elevated RD within the hippocampus. Similarly, mice with lower Y-maze alteration demonstrated decreased FA values along with increased MD and RD within the right sAMY. The consistent correlation results observed in the hippocampus across different MRI modalities suggest that changes detected by MRI within this region may reflect variations in mouse memory abilities. The MWM test is a highly effective tool for evaluating hippocampal-dependent learning and memory processes; it has been shown to rely predominantly on structural integrity, particularly within the hippocampus (Badhwar et al., 2013). Wild-type mice showed an increase of the focal volume in the hippocampus after early, long-term pioglitazone treatment. A study combining neuroimaging and behavior found that acquisition of spatial search strategies and reversal learning in the MWM depend on functional connectivity of the hippocampus (Shah et al., 2019). Our findings offer valuable insights into the relationships between structural networks and cognitive function in animal models of AD. Possible mechanisms underlying brain volume and diffusion changes in AD mouse models may depend on the effects of Aβ-deposits, such as neuronal injury or inflammatory response. A previous study has demonstrated a significant correlation between diffusion metrics and levels of Aβ and tau in the hippocampus (Falangola et al., 2022). Another has shown a complementary relationship between microstructural changes in WM and GM atrophy in a 3×Tg-AD model mouse (Bergamino et al., 2023). Building upon these previous studies, our correlation results confirm the specific involvement of the hippocampus and sAMY in pathological changes associated with AD. Structural alterations in these regions can be considered as imaging biomarkers for early diagnosis and patient stratification in AD. Future studies should employ longitudinal designs to further elucidate the mechanistic underpinnings of cognitive dysfunction in AD.

To better understand the abnormal structural imaging changes observed in the AD group, we performed immunofluorescence staining for Aβ and p-Tau (Ser404) in the whole brain. This analysis further confirms the relationship between these pathological changes and the neuroimaging and gene expression findings. Immunofluorescence analysis of the coronal brain sections revealed a significantly higher number of Aβ plaques in the hippocampus of the AD group than the control group, along with a significant difference in the expression of p-Tau. These pathological findings corroborate the MRI differences observed in the hippocampus. Based on these results, we conclude that the abnormal brain structural changes in the AD group are likely imaging manifestations of underlying pathological alterations. To gain a deeper understanding of these pathological changes and associated brain structural abnormalities, it is essential to integrate gene sequencing results into our discussion.

### Gene expression analyses

To further comprehend the neural mechanisms underlying the neuroimaging alterations in 3×Tg-AD mice, bulk RNA-seq was conducted on the hippocampus tissue. One reason this region was selected was because of the substantial level of hippocampal neurogenesis in the human brain. Previous studies have demonstrated significant functional and structural changes in the hippocampus in AD, which are likely due to impaired neurogenesis (Mu and Gage, 2011; Chiquita et al., 2019). Hippocampal neurogenesis plays a crucial role in structural plasticity and cognitive deficits observed in AD mouse models, which prompted our focus on this specific region. Another reason was because we consistently observed differences within the hippocampus between the two groups when analyzing brain imaging data using T2 and DTI modalities.

The RNA-seq analysis results in our study revealed numerous DEGs between the two groups. GO enrichment analysis demonstrated significant disparities in biological processes such as synapse structure and function, cognition, learning, and memory within the 3×Tg-AD mice group. AD is characterized by progressive memory loss and cognitive dysfunction, and age-dependent decline in neurogenesis may compromise the structure and function of the hippocampus over time, leading to susceptibility to memory impairment (Hollands et al., 2016). KEGG pathway analysis identified enrichment in 20 signaling pathways. Some of these enriched pathways are associated with nerve myelination processes, including the Wnt and MAPK signaling pathways. In a previous study, the Wnt signaling pathway inhibited oligodendrocyte differentiation and maturation while exerting a negative regulatory role in myelin development (Boccazzi et al., 2023). Mek1 is an upstream component of the MAPK signaling pathway that can enhance myelin growth (Ishii et al., 2021). Consistent with these findings, the genes significantly enriched in hippocampal regions in our study may play crucial roles in regulating myelination. These results suggest that modulation of these pathways holds potential therapeutic value for AD. Based on the results of brain imaging and brain tissue gene expression analysis, we can see that brain tissue injury in AD model mice may be affected by the level of gene expression in related tissues. This study also provides a research idea for future AD studies. Through the combined analysis of brain imaging and genetics, more comprehensive insights into the underlying neural mechanisms of AD can be gained.

### Sex as a factor in our findings

It is important to note that our study focused exclusively on male 3×Tg-AD mice, and all the differences observed in our results are based on this condition. This restriction was made to control for the potential influence of female sex hormones, which fluctuate during the estrous cycle and could significantly affect the results of the study. While single-sex studies using male animals are common in neuroscience research (Beery and Zucker, 2011), it is important to emphasize that the findings from male-only studies may not be directly applicable to female AD pathophysiology. Research has shown that hormonal fluctuations in females can influence key pathological features of AD, including β-amyloid deposition and tau phosphorylation (Babapour Mofrad and van der Flier, 2018). While our male-only design controlled for potential confounders related to cyclic hormonal fluctuations, it limits the direct translation of our results to female AD pathophysiology. Clinical evidence of sex-specific cognitive trajectories in AD further supports this distinction, as women often experience accelerated cognitive decline despite a similar or lower amyloid burden compared with men (Laws et al., 2018).

Given these considerations, we acknowledge that future studies should incorporate sex as a biological variable. Comparative analyses of male and female cohorts in this model could help uncover hormonally modulated pathways relevant to AD pathology and therapeutic development. Furthermore, including both sexes in future studies would improve the reliability and generalizability of the findings, especially in light of the clinical evidence indicating sex-specific differences in AD progression and treatment response.

## Limitations

One limitation of the present study is its focus on male 3×Tg-AD mice at 9 months of age, which may limit the generalizability of our findings to the broader patient population (Carroll et al., 2010). The small number of mice reduced the statistical power of the analysis. Furthermore, future studies should aim to investigate age-associated AD pathology to enhance our understanding of pathological attributes. Third, the DTI sequence parameters used in this study, including the b-value and the slice-to-in-plane resolution ratio, are suboptimal and require further optimization in future investigations. This aspect may account for our inability to achieve family-wise error correction during DTI data analysis. Finally, owing to the technical limitations of DTI in accurately describing intricate GM geometry and WM microstructural change, it is essential to incorporate novel acquisition methods validated for addressing these challenges. Therefore, future investigations will implement advanced DTI acquisition technologies such as multi-compartmental or non-Gaussian diffusion methods to enhance the precision of research findings.

## Conclusion

We demonstrated significant differences in brain volume and microstructural integrity between a group of 3×Tg-AD and control mice. The notable MRI differences observed in the hippocampus and sAMY exhibited a strong correlation with behavioral performance. Immunofluorescence analysis found a significantly higher number of hippocampal Aβ plaques in the AD group than the control group, while there was also significant difference in the protein expression of p-Tau. Pathological examination of the hippocampal region confirmed the results of our brain imaging. Bulk RNA-seq analysis of hippocampus tissue revealed that many DEGs were enriched in several biological processes relevant to synapse structure, cognition, learning, and memory. KEGG pathway analysis indicated that the Wnt and MAPK signaling pathways play a critical role in the nerve myelination processes in the hippocampus. These findings support the potential utilization of brain volume and diffusion metrics as biomarkers for AD pathology, which could be applied in the clinical diagnosis of AD in humans. Our results indicate a connection between regional brain changes and gene expression in AD. Combining neuroimaging techniques with RNA-seq methods can provide novel insights into the neurobiology of AD.

## Additional file:

***Additional Table 1:*** *Correlations between brain diffusion indices and behavioral performance.*

## Data Availability

*All relevant data are within the paper and its Additional files.*
